# Research on the structure and influencing factors of postgraduate students’ sense of learning gain in sports science

**DOI:** 10.3389/fpsyg.2026.1784317

**Published:** 2026-04-09

**Authors:** Jianjian Bao, Yan Lu, Kanglin Wang, Yongjia Chen, Wei Chen, Ziyi Zheng, Fen Qiu

**Affiliations:** School of Physical Education, Wuhan University of Technology, Wuhan, China

**Keywords:** influencing factors, scale development, sense of learning gain, sports science postgraduates, structural validity

## Abstract

**Objective:**

This study aimed to construct a theoretical structure of the Sense of Learning Gain (SLG) for Sports Science postgraduates, develop a standardized measurement instrument, identify its key influencing factors, and propose pathways for enhancement.

**Methods:**

Utilizing a stratified sampling method, postgraduate students from multiple provinces across China participated in an online questionnaire survey, divided into a pilot study (*n* = 302) and a formal survey (*n* = 306). The initial scale was developed through literature analysis and interviews. Exploratory Factor Analysis (EFA) was employed to determine the scale structure, followed by Confirmatory Factor Analysis (CFA) to test the model fit. Reliability and validity were assessed using Cronbach’s *α*, Composite Reliability (CR), and Average Variance Extracted (AVE). Group differences were analyzed via independent samples *t*-tests and ANOVA, and a Structural Equation Model (SEM) was constructed to examine the influencing factors.

**Results:**

(1) The final SLG Scale for Sports Science Postgraduates comprises 25 items across three dimensions: “Tangible Gains,” “Capability Enhancement,” and “Emotional Fulfillment,” with a cumulative variance explanation rate of 57.253%. (2) The formal scale demonstrated excellent reliability (Total *α* = 0.928; Subscale *α*: 0.753–0.925) and good structural validity (CFA: *χ*^2^/*df* = 2.644, RMSEA = 0.076, CFI = 0.911). (3) The overall level of SLG was relatively high (M = 3.44, SD = 0.823), with the “Tangible Gains” dimension scoring highest (M = 3.82, SD = 1.074). Significant gender (male > female, *p* < 0.05) and grade differences (first-year > second/third-year, *p* < 0.01) were observed. (4) The SEM revealed that “School Resources” (*β* = 0.457), “Social Environment” (*β* = 0.322), “Interpersonal Support” (*β* = 0.226), and “Personal Growth” (*β* = 0.196) all had significant positive effects on the SLG.

**Conclusion:**

The developed scale proves to be a reliable and valid tool for measuring the SLG among Sports Science postgraduates in China. The SLG is co-influenced by multi-level factors, and targeted optimization of the social environment, interpersonal support, school resources, and individual development can effectively enhance it.

## 1 Introduction

The concept of “Sense of Gain” (SoG), initially proposed in the context of China’s reform, emphasizes the tangible benefits and subjective satisfaction experienced by individuals. It has since permeated various fields, including education (Wang et al., 2020).

Marx and Engels emphasized in The German Ideology: “No one can accomplish anything if they do not act simultaneously for their own needs and for the organs that fulfill those needs (Engels and Marx, 2003)”. In the international theoretical system, there exist superficially similar concepts such as “satisfaction” and “sense of happiness”, but their extension definitions, internal logical structures, and measurement dimensions have essential differences from “sense of gain”. Within Maslow’s hierarchy of needs framework, the sense of fulfillment in the material dimension is concretely manifested as the realization of physiological and safety needs, primarily reflected in individuals’ tangible experiences of improved basic living conditions and stable life status (Maslow, 1943). This uniqueness is specifically manifested in three aspects: emphasizing the substantial acquisition of reform dividends in external representation, coupling the dual dimensions of material satisfaction and spiritual identity in internal structure, and highlighting the dialectical unity of social equity and individual acquisition in measurement scale. Researchers have constructed three classic models for customer satisfaction, namely SCSB, ACSI, and ECSI, to analyze the influencing factors of customer satisfaction. Among them, SCSB is the first customer satisfaction index model developed by Sweden, and the United States further adopted ACSI on this basis. With the mature application of customer satisfaction theory in the service industry, this theory has been innovatively introduced into the field of higher education quality evaluation, forming specialized evaluation dimensions such as “student satisfaction” and “educational service satisfaction”. In terms of the theoretical migration path, the international academic research on “student satisfaction” has significant theoretical affinity with the theory of sense of learning gain, and its research paradigm and methodological system provide an important reference for the study of sense of gain. Tracing the theoretical origin, the theory of customer satisfaction originated from systematic research on consumption experience and feedback behavior. Oliver’s classic definition reveals that customer satisfaction is essentially a psychological evaluation state formed after customer needs are met, reflecting their cognitive judgment on product or service quality. As an extended practice of this theory in the education field, the National Student Satisfaction Survey (NSSS) launched by the United States in 1994 pioneered the systematic quantification of college students’ satisfaction with learning experience, and its evaluation framework provided a paradigmatic foundation for subsequent research. Davis’ theoretical construction reveals that sense of happiness is essentially the result of an individual’s subjective evaluation based on a self-reference frame. When behavioral outcomes align with preset goals, individuals will produce positive emotional feedback; if goals are not achieved, negative experiences may be triggered. This internal correlation mechanism between goal achievement and sense of happiness generation provides a microscopic perspective for analyzing subjective experiences. At the theoretical connection level, Maslow’s hierarchy of needs theory provides a macroscopic analytical framework for explaining the generation mechanism of sense of gain in college students’ ideological and political education. The law of hierarchical progression of human needs it reveals not only clarifies the phased characteristics of sense of gain cultivation but also provides a theoretical basis for optimizing the matching of educational supply and demand. Studies have shown that compared with sense of happiness, sense of gain presents stronger concretization characteristics and context dependence. Its core lies in capturing the dynamic changes perceived by individuals in dimensions such as acquisition environment, content quality, experience process, realization methods, and achievement sharing within a specific time period; while sense of happiness focuses on evaluating an individual’s overall satisfaction with living conditions within a specific life cycle, with stronger stability and ultimate value orientation. This theoretical analysis shows that sense of gain is essentially a concretized expression of sense of happiness in specific practical fields, and a transformation process of subjective experience from abstract value judgment to concrete practical perception.

In higher education, “Sense of Learning Gain” (SLG), a sub-concept of SoG, is defined as an individual’s positive psychological experience derived from objective acquisitions (e.g., knowledge, skills) during the learning process, encompassing both objective and subjective attributes (Li et al., 2024). For Sports Science postgraduates, the learning process involves not only academic research (e.g., publications, projects) but also sports skill enhancement (e.g., specialized training, competition) and practical ability cultivation (e.g., teaching, event organization). This dual “academic-practical” nature makes the structure and influencing factors of their SLG particularly distinctive.

From a practical standpoint, as China advances its “Sports Power” strategy, the quality of Sports Science postgraduate training is crucial. After nearly two decades of construction and development, the professional master’s degree education in sports has achieved remarkable results, cultivating a large number of high-level applied professionals for China, covering fields such as physical education teachers, athletes, coaches, referees, as well as mass fitness and sports management. These professionals generally possess strong practical and innovative capabilities, making contributions to the development of China’s sports industry. The master’s program in sports science focuses on enhancing graduate students’ theoretical foundation in sports, interdisciplinary abilities, and professional application skills, with an emphasis on fostering their keen problem awareness and innovative ability to solve complex issues in sports practice. The high-quality development of the sports industry urgently requires the support of high-level, interdisciplinary professionals (Huang et al., 2017). As a core training component, the master’s education in sports science continues to attract significant attention from all sectors of society.

However, a perceived emphasis on “skills over literacy” and “outcomes over experience” in current education may lead to poor subjective learning experiences and diminished motivation among some students (Huang, 2023). Therefore, systematically exploring the structure and influencers of SLG is vital for optimizing training models and improving educational quality.

Regarding the research landscape, existing studies on SLG predominantly focus on undergraduate ideological-political courses or general postgraduate education, leaving a gap specifically for Sports Science. Unique dimensions like “sports skill acquisition” and “competition practice experience” are absent from current measurement frameworks. Furthermore, the impact of external factors, such as societal recognition of sports disciplines and industry development, remains unclear. Most existing studies rely on qualitative descriptions or single-dimensional measurements, lacking standardized scales and multi-level influencing factor models.

The core of SoG lies in the dialectical unity of “objective acquisition” and “subjective identification” (Tan et al., 2020). Liu and Zhou (2020) noted that SoG encompasses the fulfillment of both material (e.g., resource access) and spiritual (e.g., emotional satisfaction) needs. Structurally, Dong et al. (2019) proposed a five-dimensional structure for Chinese people’s SoG, while Wu et al. (2024) divided residents’ health SoG into four dimensions. Research on SLG originated from the subjective assessment of “learning outcomes” in education. Chen et al. (2022) developed a Postgraduate SLG scale with five dimensions. Hu and Wang (2019) proposed a three-dimensional structure for undergraduate SLG. In sports, research mainly focuses on undergraduate course SoG, often centering on the single dimension of “skill improvement,” neglecting the integration of academia and practice at the postgraduate level. Sports Science postgraduate education has a dual “academic” and “practical” orientation (Huang et al., 2017). This specificity dictates that their SLG should include not only “knowledge acquisition” and “capability enhancement” but also unique dimensions like “sports skill progress” and “satisfaction from practice/competition” (Fang et al., 2018). External factors like societal recognition and employment prospects in the sports industry may also influence SLG by affecting professional identity (Huang, 2023).

Our research adopts a comparative analysis method to conduct theoretical analysis on the two core concepts of “sense of gain” and “sense of happiness”, aiming to more accurately define the conceptual boundaries and connotative characteristics of sense of gain. The study found that both belong to the category of subjective psychological experience but have significant differences: sense of happiness focuses on an individual’s subjective judgment of life satisfaction, with its evaluation dimensions having ultimate value; sense of gain emphasizes the universal sharing of development achievements through systematic arrangements such as policy design, institutional optimization, and practical implementation in the entire process of reform and development, and this sharing nature is its core characteristic distinguishing it from sense of happiness. The interaction between this objective acquisition and subjective perception makes sense of gain more concrete and systematic than sense of happiness in connotative dimension, forming a dual logic of practice-experience.

This study aims to construct the theoretical structure of Sports Science postgraduates’ SLG and develop a standardized measurement scale with good reliability and validity, analyze the overall level of SLG and its group differences across gender, grade, and major, identify the key influencing factors of SLG and build a multi-level model, as well as propose targeted enhancement pathways based on these influencing factors.

## 2 Methods

### 2.1 Participants and sampling

A stratified sampling method was employed to recruit postgraduate students majoring in Sports Science from universities across several provinces in China, including Hubei, Henan, Hunan, and Shanghai. The survey was conducted online and consisted of two phases: a pilot study and a formal survey.

#### 2.1.1 Pilot survey participants

A total of 315 questionnaires were distributed, with 302 valid responses retrieved, yielding an effective response rate of 95.87%. The sample comprised 159 males (52.65%) and 138 females (47.35%). In terms of grade distribution, there were 80 first-year students (26.49%), 83 s-year students (27.48%), 93 third-year students (30.79%), and 46 recent graduates (15.23%). Regarding majors, the sample included 72 students from Sports Humanities and Sociology (23.84%), 68 from Human Movement Science (22.52%), 112 from Sports Education and Training (37.09%), and 50 from Traditional Ethnic Sports (16.56%).

#### 2.1.2 Formal survey participants

For the formal survey, 320 questionnaires were distributed, resulting in 306 valid responses and an effective response rate of 95.63%. This sample included 164 males (53.59%) and 142 females (46.41%). The grade distribution was as follows: 82 first-year students (26.80%), 85 s-year students (27.78%), 91 third-year students (29.74%), and 48 recent graduates (15.69%). The major distribution was: 75 from Sports Humanities and Sociology (24.51%), 70 from Human Movement Science (22.88%), 115 from Sports Education and Training (37.58%), and 46 from Traditional Ethnic Sports (15.03%).

### 2.2 Instrument development

#### 2.2.1 Initial scale development

Item Pool Generation: The initial item pool was developed through a dual approach:

Literature Analysis: A comprehensive review of existing research on SoG and SLG was conducted to identify core dimensions such as “knowledge acquisition,” “capability enhancement,” and “emotional fulfillment.” This was integrated with the unique characteristics of Sports Science postgraduates (e.g., “sports skill advancement,” “competition practice”), resulting in 35 preliminary items.

Interviews: Semi-structured interviews were carried out with 23 Sports Science postgraduates. The interview protocol included questions such as, “In what aspects are your ‘gains’ reflected during your postgraduate stage?” and “What factors influence your SLG?” Based on the interview results, 8 additional items were generated, culminating in an initial pool of 43 items.

Content Validity Assessment: Three experts in sports psychology (two professors and one associate professor) and five Sports Science postgraduates were invited to evaluate the initial items. They rated the items on “clarity,” “relevance,” and “representativeness” using a 1–4 point scale (4 being optimal). The Item-Content Validity Index (I-CVI) ranged from 0.83 to 1.00, and the Scale-Content Validity Index (S-CVI) was 0.92, indicating good content validity. Following expert suggestions, two ambiguously phrased items were deleted, resulting in 41 items retained for the pilot test.

#### 2.2.2 Pilot scale

The 41-item pilot scale utilized a Likert 5 scale (1 = “Strongly Disagree,” 5 = “Strongly Agree”).

#### 2.2.3 Influencing factors questionnaire

Based on literature analysis and interview findings, a preliminary Influencing Factors Questionnaire was constructed. It hypothesized four latent dimensions: “Social Environment” “Interpersonal Support” “School Resources” and “Personal Growth.” Each dimension contained 6 items, totaling 24 items, all measured on a Likert 5 scale.

### 2.3 Procedure

The research procedure involved three main stages:

Pilot survey (March–April 2024): the initial scale was administered. Item analysis (Critical Ratio test, Corrected Item-Total Correlation) and Exploratory Factor Analysis (EFA) were performed to screen and refine the items, forming the formal scale.

Formal survey (May–June 2024): the formal SLG Scale and the Influencing Factors Questionnaire were distributed to collect data for reliability and validity tests, group difference analysis, and model construction.

Data consolidation and analysis (July–August 2024): data analysis was conducted using SPSS 23.0 and AMOS 21.0.

### 2.4 Data analysis

The following statistical methods were employed:

Item analysis: items were screened using the Critical Ratio (CR) > 3and Corrected Item-Total Correlation (CITC) > 0.3. Items failing these criteria were deleted.

Exploratory factor analysis (EFA): principal Component Analysis with Varimax rotation was used. Factors were retained based on Eigenvalue >1 and factor loading >0.4.

Confirmatory factor analysis (CFA): the Maximum Likelihood method was used to assess model fit. Fit indices included *χ*^2^/*df* (<3 considered good), *RMSEA* (<0.08), CFI (>0.9), TLI (>0.9), and IFI (>0.9).

Reliability analysis: internal consistency was evaluated using Cronbach’s *α*, with *α* > 0.7 considered acceptable.

Validity analysis: convergent validity was assessed via Composite Reliability (CR) > 0.6 and Average Variance Extracted (AVE) > 0.5.

Group difference analysis: independent samples t-test was used for gender differences. One-way ANOVA or Welch’s test was employed for grade and major differences.

Influencing factors model: structural equation modeling (SEM) was applied to examine the path relationships between the influencing factors and the SLG, testing model fit and path coefficient significance.

## 3 Results

### 3.1 Development and validation of the sense of learning gain scale

#### 3.1.1 Item analysis

Critical ratio (CR) test: the pilot sample was ranked by total scale score, with the top 27% designated as the high-score group (*n* = 82) and the bottom 27% as the low-score group (*n* = 82). Independent samples t-test results indicated that all 41 items had CR values > 3 (*p* < 0.001), demonstrating good item discrimination.

Corrected item-total correlation (CITC) analysis: the CITC values for all items exceeded 0.3 (range: 0.389–0.877). Consequently, no items were deleted based on this criterion.

#### 3.1.2 Exploratory factor analysis (EFA)

Sample adequacy: the Kaiser–Meyer–Olkin (KMO) measure was 0.936 (>0.9), and Bartlett’s test of sphericity was significant (*χ*^2^ = 9880.948, *df* = 820, *p* < 0.001), confirming the suitability of the data for EFA.

Factor Extraction and Item Screening: Principal Component Analysis with Varimax rotation extracted three factors with eigenvalues greater than 1, cumulatively explaining 57.253% of the variance (Factor 1: 37.937%, Factor 2: 11.163%, Factor 3: 8.253%). Following the criterion of factor loading > 0.4 and considering cross-loadings, 16 items were removed, resulting in a final 25-item scale. Displays the specific factor loadings of the final retained items under each factor (Table 1).

**Table 1:** Specific factor loadings.

Dimension	Number of questions	Item	Content	Load
Actual acquisition	10	T19	The professional study at the postgraduate level gave me the opportunity to obtain a scholarship.	0.635
		T32	Communication with my tutor can improve my learning and research quality.	0.733
		T26	The study of graduate degree can enrich my knowledge system.	0.801
		T23	Studying for a master’s degree in sports can increase my employment opportunities.	0.607
		T35	The study of the research stage makes me deeply understand the Chinese sports spirit.	0.750
		T30	Participating in sports activities during the postgraduate stage has helped me maintain a good physical and mental state.	0.751
		T28	The study and practice in graduate school have expanded my interpersonal relationship.	0.772
		T34	The study of professional skills has broadened my interests.	0.702
		T29	The study of sports professional knowledge in graduate school is helpful to my healthy life.	0.767
		T38	Professional knowledge learning and academic training help me to publish academic papers.	0.726
Ability enhancement	8	T20	The study of graduate degree has improved my ability of independent thinking.	0.848
		T49	The process of sports science research improves my information retrieval ability.	0.746
		T48	The study of postgraduate degree has improved my self-control.	0.819
		T46	Studying for a master’s degree in sports can improve my core competitiveness.	0.844
		T45	The study of postgraduate level has improved my ability to solve the problems of sports practice.	0.808
		T39	In the process of sports scientific research, my ability of critical thinking and logical thinking is improved.	0.511
		T42	The study of postgraduate level has improved my ability of organizing sports events.	0.670
		T41	The postgraduate study has improved my ability of physical education teaching.	0.734
Emotional fulfillment	7	T2	In the professional sport skill learning, the sport skill level is improved and I feel happy.	0.595
		T5	I will be satisfied if I can apply the sports professional knowledge I have learned in the postgraduate study.	0.561
		T4	After mastering the professional knowledge of physical education, I have a sense of fulfillment.	0.684
		T10	I feel successful when I can achieve the goal of sports research.	0.486
		T1	I feel very satisfied that my academic paper has been published in the sports journal.	0.716
		T8	I get excited when I get a place in a sports competition.	0.660
		T12	The improvement of sports technical level makes me have a sense of achievement in practice.	0.577

Factor naming:

Factor 1 (10 items): included items such as “receiving scholarships,” “publishing academic papers,” and “enriching knowledge base,” focusing on objective learning outcomes. It was named “Tangible Gains.” The final retention factor loading range was 0.607–0.801.

Factor 2 (8 items): Included items like “improving independent thinking ability,” “enhancing event organization skills,” and “strengthening research skills,” focusing on competency development. It was named “Capability Enhancement.” The final retention factor loading range was 0.511–0.848.

Factor 3 (7 items): included items such as “pleasure from improving sports skills,” “satisfaction from paper publication,” and “sense of achievement from winning competitions,” focusing on subjective emotional experiences. It was named “Emotional Fulfillment.” The final retention factor loading range was 0.4867–0.716.

### 3.2 Reliability and validity of the formal scale

#### 3.2.1 Reliability analysis

Internal consistency, measured by Cronbach’s *α*, was excellent for the total scale (*α* = 0.928) and for each dimension: “Tangible Gains” (*α* = 0.919), “Capability Enhancement” (*α* = 0.925), and “Emotional Fulfillment” (*α* = 0.753).

#### 3.2.2 Validity analysis

Structural validity (CFA): confirmatory factor analysis was performed on the formal survey data (*n* = 306) for the three-dimensional model. The initial model fit was: *χ*^2^/*df* = 3.106, RMSEA = 0.084, CFI = 0.885, TLI = 0.873, IFI = 0.886. Building upon the research of Wen et al. (2004), this study incorporates covariance into the framework using the fitting index and chi-square criterion (Table 2).

**Table 2:** Model correction index.

Residual term	M.I	Par change
e16↔e17	72.317	0.206
e17↔e18	39.216	0.094

After adding covariance’s between error terms e16 & e17 and e17 & e18 based on modification indices, the fit improved significantly: *χ*^2^/*df* = 2.644 (<3), RMSEA = 0.076 (<0.08), CFI = 0.911 (>0.9), TLI = 0.901 (>0.9), IFI = 0.912 (>0.9). These indices indicate a good model fit and strong structural validity (Table 3).

**Table 3:** Confirmatory factor analysis model fit indices.

Model	*χ*^2^/*df*	RMSEA	CFI	TLI	IFI
Initial 3-factor	3.106	0.084	0.885	0.873	0.886
Revised 3-factor	2.644	0.076	0.911	0.901	0.912
Recommended value	<3	<0.08	>0.9	>0.9	>0.9

Convergent validity: the composite reliability (*CR*) and Average Variance Extracted (AVE) values were calculated. “Tangible Gains” (CR = 0.921, AVE = 0.583), “Capability Enhancement” (CR = 0.927, AVE = 0.591), and “Emotional Fulfillment” (CR = 0.762, AVE = 0.431) all demonstrated acceptable convergent validity, noting that AVE for Emotional Fulfillment, while below 0.5, is considered acceptable given its CR value (Table 4).

**Table 4:** Convergent validity and reliability of the formal scale.

Dimension	No. of items	CR	AVE	Cronbach’s *α*
Tangible gains	10	0.921	0.583	0.919
Capability enhancement	8	0.927	0.591	0.925
Emotional fulfillment	7	0.762	0.431	0.753
Total scale	25	—	—	0.928

Discriminant Validity: The correlations between the three dimensions (ranging from 0.389 to 0.629, *p* < 0.01) were all lower than the square root of the AVE for each dimension and their correlations with the total scale score (ranging from 0.674 to 0.877, *p* < 0.01), indicating good discriminant validity (Table 5).

**Table 5:** Discriminant validity: correlations between dimensions and total score.

Dimension	Tangible gains	Capability enhancement	Emotional fulfillment	Total scale
Tangible gains	1.000	0.629**	0.389**	0.877**
Capability enhancement	—	1.000	0.397**	0.838**
Emotional fulfillment	—	—	1.000	0.674**

***p < 0.001, **p < 0.01, *p < 0.05.

### 3.3 Level of sense of learning gain and group differences

#### 3.3.1 Overall level

Descriptive statistics revealed that the total score and scores for all three dimensions of the SLG were above the scale midpoint of 3, indicating a relatively high overall level. The total mean score was 3.44 (SD = 0.823). Among the dimensions, “Tangible Gains” had the highest mean score (M = 3.82, SD = 1.074), followed by “Capability Enhancement” (M = 3.32, SD = 0.986), and “Emotional Fulfillment” had the lowest mean score (M = 3.03, SD = 0.939) (Table 6).

**Table 6:** Descriptive statistics for SLG (*N* = 306).

Dimension	Mean (M)	Standard deviation (SD)	Min	Max
Tangible gains	3.82	1.074	1.10	5.00
Capability enhancement	3.32	0.986	1.25	4.75
Emotional fulfillment	3.03	0.939	1.00	5.00
Total scale	3.44	0.823	1.16	4.88

#### 3.3.2 Group difference analysis

Gender Differences: Independent samples t-tests showed that male postgraduates scored significantly higher than females on “Tangible Gains” (*t* = 2.15, *p* = 0.032), “Capability Enhancement” (*t* = 4.16, *p* < 0.001), “Emotional Fulfillment” (*t* = 2.57, *p* = 0.011), and the total score (*t* = 3.45, *p* = 0.001) (Table 7).

**Table 7:** SLG: gender differences (M ± SD).

Dimension	Gender	M ± SD	*t*	*p*
Tangible gains	Male	3.94 ± 0.98	2.15	0.032
	Female	3.68 ± 1.16	—	—
Capability enhancement	Male	3.23 ± 0.92	4.16	<0.001
	Female	2.80 ± 0.90	—	—
Emotional fulfillment	Male	3.45 ± 0.97	2.57	0.011
	Female	3.16 ± 0.99	—	—
Total scale	Male	3.58 ± 0.77	3.45	0.001
	Female	3.26 ± 0.85	—	—

Grade differences: tests of homogeneity of variance indicated that “Capability Enhancement” and “Emotional Fulfillment” met the assumption (p > 0.05), thus ANOVA was used. “Tangible Gains” and the total score violated this assumption (*p* < 0.05), so Welch’s test was applied. Results indicated significant differences across grade levels in all dimensions and the total score (*p* < 0.01). Post-hoc comparisons (LSD/Tamhane’s T2) revealed: (1) For “Tangible Gains,” first-year students scored significantly higher than third-year students (*p* = 0.012), and graduates scored significantly lower than both second-year (*p* = 0.024) and third-year students (*p* = 0.004). (2) For “Capability Enhancement” and “Emotional Fulfillment,” first-year students scored significantly higher than both second-year and third-year students (all *p* < 0.001). Graduates scored significantly lower than second-year and third-year students on these dimensions (all *p* < 0.001). The primary reasons for differences in learning gains across grade levels include variations in learning stages and tasks, mentorship factors, school resources and support, individual factors, interpersonal relationships and teamwork, as well as academic pressure and mental health (Lu, 2021) (Table 8).

**Table 8:** SLG: grade differences (M ± SD).

Dimension	1st year (*n* = 82)	2nd year (*n* = 85)	3rd year (*n* = 91)	Graduates (*n* = 48)	*F*	*p*
Tangible gains	3.98 ± 1.02	3.85 ± 1.05	3.62 ± 1.11	3.71 ± 1.08	6.197	0.001
Capability Enh.	3.56 ± 0.95	3.21 ± 0.97	3.18 ± 0.99	3.25 ± 0.96	13.443	<0.001
Emotional Fulf.	3.28 ± 0.92	2.98 ± 0.94	2.87 ± 0.96	3.01 ± 0.93	12.446	<0.001
Total scale	3.61 ± 0.80	3.42 ± 0.83	3.29 ± 0.85	3.35 ± 0.81	15.315	<0.001

Major differences: one-way ANOVA revealed no significant differences in “Tangible Gains” (*F* = 0.063, *p* = 0.979), “Capability Enhancement” (*F* = 1.753, *p* = 0.156), “Emotional Fulfillment” (*F* = 0.323, *p* = 0.809), or the total score (*F* = 0.289, *p* = 0.834) among the four sports science majors.

### 3.4 Influencing factors mode

#### 3.4.1 Initial model and measurement model check

A hypothesized model was proposed where four latent variables—“Social Environment,” “Interpersonal Support,” “School Resources,” and “Personal Growth”—positively influence the SLG (H1-H4).

The Influencing Factors Questionnaire itself demonstrated good reliability (Cronbach’s *α*: 0.876–0.905; Total *α* = 0.931) and structural validity (CFA: *χ*^2^/*df* = 2.121, RMSEA = 0.061, CFI = 0.949, TLI = 0.943, IFI = 0.950).

#### 3.4.2 Structural model and path analysis

Structural Equation Modeling (SEM) was used to test the path relationships. The initial model fit was: *χ*^2^/*df* = 2.520, *RMSEA* = 0.071, CFI = 0.892, TLI = 0.886, IFI = 0.892. After adding covariance’s between error terms e5 & e6, e10 & e11, and e44 & e45 based on modification indices, the revised model fit met acceptable standards: *χ*^2^/*df* = 2.386 (<3), RMSEA = 0.067 (<0.08), CFI = 0.903 (>0.9), TLI = 0.896 (approaching 0.9), IFI = 0.902 (>0.9).

Path analysis results confirmed all four hypotheses (Table 9):

**Table 9:** Path analysis results of the influencing factors model.

Path (SLG ←)	Unstd.estimate	S.E.	C.R.	*p*	*β*
Social environment	1.000	—	—	—	0.322
Interpersonal support	0.748	0.209	3.589	<0.001	0.226
School resources	2.514	0.533	4.716	<0.001	0.457
Personal growth	0.731	0.260	2.805	0.005	0.196

“School Resources” had the strongest significant positive effect on SLG (*β* = 0.457, *p* < 0.001), supporting H3.

“Social Environment” had a significant positive effect (*β* = 0.322, *p* < 0.001), supporting H1.

“Interpersonal Support” had a significant positive effect (*β* = 0.226, *p* < 0.001), supporting H2.

“Personal Growth” had a significant positive effect (*β* = 0.196, *p* = 0.005), supporting H4.

## 4 Discussion

### 4.1 The structure of sense of learning gain in sports science postgraduates

This study established a three-dimensional structure—“Tangible Gains,” “Capability Enhancement,” and “Emotional Fulfillment”—for the SLG among Sports Science postgraduates. This structure aligns with the general framework proposed by Hu and Wang (2019) while incorporating the unique “academic-practical” duality of the discipline (Huang, 2023). The “Tangible Gains” dimension, encompassing items like publishing papers and winning competitions, reflects the concrete outcomes valued in both academic and practical tracks. Actual achievement constitutes a vital component of learning fulfillment, directly reflecting the substantive progress and gains attained by graduate students during their academic process (Bie and Tao, 2009). The “Capability Enhancement” dimension, including sport-specific skills like teaching and technical analysis, highlights the core competencies required for success in this field (Fang et al., 2018). The “Emotional Fulfillment” dimension, particularly items related to the joy of skill improvement, captures a distinct aspect of the subjective experience for Sports Science students, differentiating it from the purely academic satisfaction often studied in other contexts (Liang, 2023). Psychometrically, the scale demonstrated excellent reliability and validity, establishing it as a robust tool for future research.

### 4.2 Level and differences in sense of learning gain

According to the motivational theory in educational psychology, a strong academic atmosphere can stimulate graduate students’ academic interest and exploratory desire (Barney, 1991). The relatively high overall level of SLG, particularly in “Tangible Gains,” likely stems from the explicit reward structures (e.g., scholarships, publication requirements) and the immediate, observable feedback inherent in sports skill development (Huang et al., 2017). Conversely, the lower score in “Emotional Fulfillment” may indicate that deeper needs for professional identity, societal recognition, and confidence in career prospects are not being fully met, potentially overshadowed by academic pressures and employment anxieties (Wang and Zhao, 2024).

The significant gender difference, with males reporting higher SLG across all dimensions, is consistent with previous findings (Li et al., 2018). Potential explanations include physiological advantages in certain sports, societal stereotypes favoring male achievement in athletic and sometimes academic spheres of sports science (Johnson and Smith, 2015; Eagly and Wood, 2012), and potentially more optimistic employment prospects for males in roles like coaching (Huang, 2023).

The observed grade difference, characterized by a peak in the first year followed by a decline, aligns with the typical postgraduate trajectory. The initial “honeymoon phase” of new knowledge and skills gives way to the pressures of thesis research and job hunting in later years, with a potential slight recovery upon completion as students reflect on their cumulative achievements (Wang, 2019; Bai and Chu, 2024). The lack of significant differences among majors suggests a shared core experience and common challenges across the various sub-fields of Sports Science in China (Fang et al., 2018).

### 4.3 Mechanism of influencing factors

Analysis of mean scores reveals that male sports science graduate students consistently outperform their female counterparts across three evaluation dimensions—actual achievement, skill enhancement, and emotional fulfillment—as well as in overall scores. The most striking gender disparity emerges in skill enhancement, where the score difference is particularly pronounced. This phenomenon may stem from physiological differences and personality traits between genders, a characteristic that becomes more pronounced in sports science—a discipline traditionally favored by males. The consistent gender gap in male students’ multidimensional scores aligns with previous research findings (Li et al., 2018). Contributing factors include both physiological advantages in certain sports and societal stereotypes about male achievements in sports (Johnson and Smith, 2015; Eagly and Wood, 2012). Additionally, the more optimistic career prospects for men in coaching and other sports-related positions further reinforce this gender disparity (Huang, 2023).

The significant effect of the “Social Environment” (*β* = 0.322) highlights the importance of macro-level context. National strategies like “Sports Power” and the growth of the sports industry enhance the perceived value of the discipline and improve career optimism, thereby indirectly boosting SLG (Wang and Zhao, 2024).

“Interpersonal Support” (*β* = 0.226) acts as a crucial intermediary. Effective mentoring provides both instrumental guidance (enhancing tangible gains and capabilities) and emotional sustenance (Kram, 1985). Peer collaboration and family understanding further contribute to a supportive network that fosters learning and well-being (Zhang et al., 2023).

Our study examined graduate students across different sports science disciplines. The results indicate no significant differences in the dimensions or total scores of learning fulfillment among sports science students (*p* > 0.05), demonstrating that the discipline does not affect their learning fulfillment.

Finally, “Personal Growth” (*β* = 0.196) represents the essential internal driver. Strong learning motivation, self-efficacy built on past successes, and genuine academic interest fuel the proactive pursuit of gains and enhance the emotional reward derived from achievements (Ryan and Deci, 2017; Bandura, 1997).

### 4.4 Implications for enhancement

The identified factors provide a clear, multi-level framework for intervention. The proposed pathways — optimizing the social environment (for instance, mass media can leverage its extensive dissemination capabilities to improve and optimize the social environment (Chen et al., 2024)), enhancing school resources (for instance, Encourage enterprises to establish long-term and stable cooperative relationships with universities to jointly create internship bases, providing abundant practical positions for postgraduate students in sports science. (Gu et al., 2024; Xiao et al., 2023), strengthening interpersonal support(When individuals perceive their own progress or advantages through comparison with others, they develop a sense of achievement and positive self-perception. Conversely, if they recognize gaps, it also motivates them to strive for improvement, thereby enhancing self-growth and learning satisfaction during the learning process (Suls and Wills, 1991)), and fostering personal development (for instance, based on constructivist learning theory, a training model that integrates theory with practice and equally emphasizes academic research and applied practice is adopted (Van Wormer and Besthorn, 2017; Ji and Wang, 2019)) — are directly targeted, systematic, and actionable for educational institutions and policymakers (Song et al., 2024). This holistic approach is likely more effective than interventions focusing on a single aspect. Finally, enhancing the sense of hope among postgraduate students in sports science. The sense of learning hope is not merely a subjective feeling but rather a relatively calm and objective judgment. Postgraduate students in sports science have a clear understanding of their current learning status and future academic development prospects (Meng et al., 2023).

## 5 Conclusion

This study successfully developed a reliable and valid 25-item scale measuring the three-dimensional SLG of Sports Science postgraduates. The overall SLG level is relatively high but varies by dimension, gender, and grade. It is significantly influenced by school resources, social environment, interpersonal support, and personal growth, suggesting the need for holistic enhancement strategies.

## 6 Research limitations and future prospects

This study has four significant limitations, which exert specific and critical impacts on the strength, direction, and generalizability of the research conclusions:

First, the imbalanced regional representativeness of the sample. Although the study claims to cover multiple provinces, most samples are concentrated in Hubei Province, with an extremely low proportion of samples from universities in western, northeastern, and other regions. This regional distribution bias directly weakens the generalizability of the conclusions: there are significant differences in the development level of the sports industry, local policy support for sports disciplines, and the allocation of sports resources in universities across different regions. Such differences may lead to heterogeneity in the impact intensity of “social environment” and “school resources” on the sense of learning gain (SLG) across regions. For instance, universities in western China may have a weaker positive driving effect of the “social environment” than the results of this study due to relatively scarce sports industry resources and limited investment in scientific research funds, while the marginal effect of “school resources” may be higher.

Second, the study was designed as a cross-sectional study. The study analyzes the current status and influencing factors of SLG through data collection at a single time point, failing to dynamically capture its long-term evolutionary characteristics and the phased changes of influencing factors. Graduate students have different core needs and pressures at different learning stages: freshmen are in an “exploratory period” and rely more on school resources and interpersonal support; second and third-year students enter an “intensive period,” where the pressure of paper publication and research projects may highlight the internal driving role of “personal growth”; and graduating students face a “transition period,” where factors such as employment prospects and industry recognition in the social environment may have a significantly enhanced impact on SLG.

Third, the single type of institutions from which the sample is sourced. Most samples in this study are from comprehensive universities, with an extremely low proportion of samples from professional sports universities. However, there are essential differences in the training objectives, resource allocation, and teaching models between these two types of institutions: comprehensive universities focus more on cultivating academic research capabilities, with a higher proportion of scientific research platforms and academic exchange opportunities in school resources; professional sports universities, on the other hand, focus on practical skills and industry connection, with more prominent industry resources and practical base support in the social environment. This sample structure bias makes it difficult for the research results to fully reflect the overall situation of postgraduate education in sports disciplines in China. For example, the “ability improvement” dimension of students in professional sports universities may be more driven by industry needs in the social environment, while students in comprehensive universities may rely more on the academic resources of the school.

Fourth, the average extracted variance (AVE) of the ‘emotional satisfaction’ dimension in this study was 0.431, which is below the commonly used threshold of 0.5. Although the composite reliability (CR) reached 0.762, meeting the acceptable level, it still indicates insufficient convergent validity for this dimension. This issue may weaken the discriminant validity between the ‘emotional satisfaction’ dimension and other dimensions.

Future research can be further advanced in four aspects to make up for the above limitations:

(1) Expand the sample scope and optimize the sample structure, focusing on increasing samples from universities in western and northeastern regions as well as professional sports universities to ensure the representativeness of regions and institution types. Verify the applicability of the influencing factor model in different groups through multi-group confirmatory factor analysis to improve the external validity of the conclusions;
(2) Adopt a longitudinal tracking design to conduct long-term data collection on an annual basis, construct a latent growth model, explore the phased evolutionary trajectory of SLG and the dynamic weight changes of influencing factors, and reveal its long-term development laws;
(3) Combine qualitative research methods, such as in-depth interviews and focus group discussions, to explore the core demands and influencing mechanisms of SLG among postgraduates in different regions, different types of institutions, and different learning stages, so as to provide richer empirical basis for formulating more targeted improvement strategies;
(4) Revise the measurement items of the “emotional satisfaction” dimension, optimize the item expression and content coverage through supplementary interviews and expert reviews to improve the convergent validity of this dimension. Meanwhile, ensure the psychometric quality of the scale through cross-validation with diverse samples, providing a more reliable measurement tool for subsequent research.

## Data Availability

The original contributions presented in the study are included in the article/Supplementary material, further inquiries can be directed to the corresponding author.
