# Supplementary material for: Research on the structure and influencing factors of postgraduate students’ sense of learning gain in sports science
Source: Front Psychol. 2026 Apr 9;17:1784317. doi: 10.3389/fpsyg.2026.1784317 (PMC13102843; doi:10.3389/fpsyg.2026.1784317)
Supplement: Supplementary file 1 [file Supplementary_file_1.docx]

**Questionnaire on the Influencing Factors of Sports Science Postgraduates' Sense of Learning Gain**

**The following statements describe specific situations of individuals during the postgraduate study in sports science. Please select the most appropriate option according to your actual situation.**

| Statements | 1 | 2 | 3 | 4 | 5 |
| --- | --- | --- | --- | --- | --- |
| 1. The social recognition of sports majors affects my sense of learning gain. | □ | □ | □ | □ | □ |
| 2. The positive social sports atmosphere of national fitness affects my sense of learning gain. | □ | □ | □ | □ | □ |
| 3. The social demand for sports talents affects my sense of learning gain. | □ | □ | □ | □ | □ |
| 4. The favorable development trend of the sports discipline frontier affects my sense of learning gain. | □ | □ | □ | □ | □ |
| 5. The application and development of modern information technology in sports majors affect my sense of learning gain. | □ | □ | □ | □ | □ |
| 6. The development of the sports industry and promising employment prospects affect my sense of learning gain. | □ | □ | □ | □ | □ |
| 7. If my supervisor provides targeted guidance and suggestions during postgraduate studies, my sense of learning gain will increase accordingly. | □ | □ | □ | □ | □ |
| 8. If I receive recognition from others during postgraduate studies, my sense of learning gain will increase accordingly. | □ | □ | □ | □ | □ |
| 9. If there is a high degree of matching between teachers and students in academic interests and research directions during postgraduate studies, my sense of learning gain will increase accordingly. | □ | □ | □ | □ | □ |
| 10. If my academic and research achievements are better than those of my classmates during postgraduate studies, my sense of learning gain will increase accordingly. | □ | □ | □ | □ | □ |
| 11. If I establish good interpersonal relationships with classmates and supervisors during postgraduate studies, my sense of learning gain will increase accordingly. | □ | □ | □ | □ | □ |
| 12. If my supervisor helps solve puzzles in learning and research during postgraduate studies, my sense of learning gain will increase accordingly. | □ | □ | □ | □ | □ |
| 13. A reasonable curriculum design by the university is conducive to improving my sense of learning gain. | □ | □ | □ | □ | □ |
| 14. If the university provides diverse academic exchange platforms during postgraduate studies, my sense of learning gain will increase accordingly. | □ | □ | □ | □ | □ |
| 15. If the university attaches importance to sports culture during postgraduate studies, my sense of learning gain will increase accordingly. | □ | □ | □ | □ | □ |
| 16. A university curriculum that meets my learning needs is conducive to improving my sense of learning gain. | □ | □ | □ | □ | □ |
| 17. If the university’s sports venues and facilities are relatively complete during postgraduate studies, my sense of learning gain will increase accordingly. | □ | □ | □ | □ | □ |
| 18. A strong academic atmosphere can stimulate my academic interest and enthusiasm, which is conducive to improving my sense of learning gain. | □ | □ | □ | □ | □ |
| 19. If I obtain national or university scholarships during postgraduate studies, my sense of learning gain will increase accordingly. | □ | □ | □ | □ | □ |
| 20. If my sports technical level improves during postgraduate studies, my sense of learning gain will increase accordingly. | □ | □ | □ | □ | □ |
| 21. If I have clear goals for postgraduate studies during this period, my sense of learning gain will increase accordingly. | □ | □ | □ | □ | □ |
| 22. If I receive the title of "Outstanding Graduate" upon completing postgraduate studies, my sense of learning gain will increase accordingly. | □ | □ | □ | □ | □ |
| 23. If I have a strong pursuit of academic achievement during postgraduate studies, my sense of learning gain will increase accordingly. | □ | □ | □ | □ | □ |
| 24. If I publish journal papers during postgraduate studies, my sense of learning gain will increase accordingly. | □ | □ | □ | □ | □ |
